# A Functional Polymorphism in HIF-3α Is Related to an Increased Risk of Ischemic Stroke

**DOI:** 10.1007/s12031-020-01728-z

**Published:** 2020-11-23

**Authors:** Xi-Xi Gu, Zhuan-Zhi Tang, Yong-Ling He, Zhi-Neng Zeng, Wu-Xiang Shi, Yong-Chao Qiao, Ye-Sheng Wei

**Affiliations:** 1 Department of Clinical Laboratory, Affiliated Hospital of Guilin Medical University, Guilin, Guangxi China; 2 Guilin Medical University, Guilin, Guangxi China

**Keywords:** Ischemic stroke, Hypoxia-inducible factor-3α, Gene, Polymorphism

## Abstract

Hypoxia-inducible factor-3α (*HIF-3α*), a member of HIF family, can mediate adaptive responses to low oxygen and ischemia. It is believed that HIF plays crucial roles in stroke-related diseases. However, there are no reports on the association between HIF-3α genetic variants and ischemic stroke (IS) susceptibility. Therefore, we examined the association between *HIF-*3α gene polymorphisms (rs3826795, rs2235095, and rs3764609) and IS risk. The study population included 302 controls and 310 patients with ischemic stroke. Three polymorphisms in *HIF-*3α (rs3826795, rs2235095, and rs3764609) were genotyped using SNPscan technique. Our study showed a strong association of rs3826795 in HIF-3α with the risk of IS. The genotype and allele frequencies were shown to differ between the two groups. The rs3826795 in an intron of *HIF-3α* was related to a prominent increased IS risk (AA vs GG adjusted odd ratio [OR], 2.21; 95% confidence intervals [95% CI], 1.10–4.44; *P* = 0.03; AA vs AG/GG OR = 1.74, 95% CI, 1.02–2.97, *P* = 0.04; A vs G OR = 1.48, 95% CI, 1.05–2.07, *P* = 0.02). Logistic regression analysis suggested that rs3826795 posed a risk factor for IS in addition to common factors. Furthermore, when compared to controls, increased levels of homocysteic acid and level of non-esterified fatty acid were found in the cases (*P* < 0.01). However, no significant association was found between rs2235095 or rs3264609 and IS risk. These findings indicated that the rs3826795 polymorphism may be a potential target for predicting the risk of IS.

## Introduction

Stroke is considered to be the main cause of death and permanent disability worldwide, causing serious economic and social impacts (Amp and Wilkins 2017; Brand et al. 2009; Susan a. Randolph 2016). Likewise, both the life quality and expectancy of life in patients are seriously influenced. Ischemic stroke (IS) is a more common type, and it accounts for approximately 85% of total cases (Rosamond et al. 2007). Previously, atherosclerosis has been found to play a major role in the IS process (Amarenco et al. 2004). Moreover, the occurrence and development of IS are also affected by a host of risk factors, including environmental and genetic risk factors (Feigin et al. 2009; Fonseca and Ferro 2015; Guzik and Bushnell 2017; Hankey 2014).

The hypoxia-inducible factor-α (HIF-α) family has 3 different members (Duan 2016). However, it is well known more about HIF-1α and HIF-2α than HIF-3α due to multiple splicing variants of HIF-3α. Hara and his team discovered human HIF-3α (hHIF-3α) in 2001 (Hara et al. 2001). The *HIF-3α* gene is located in 19q13.32 with a full length of 43 kb, and contains 8 alternative splicing and 19 introns (Pasanen et al. 2010). It is thought that HIF-3α is a negative regulator by competing with HIF-1α/HIF-2α for common HIF-β (Duan 2016). HIF participates actively in angiogenesis, erythropoiesis, cell cycle regulation, metabolic reprograming, and tumorigenic changes (Greer et al. 2012; Mylonis and Simos 2019; Shay and Celeste Simon 2012). Some *HIF-3α* target genes have been identified and contribute to various diseases, such as idiopathic pulmonary fibrosis (Aquino-Galvez et al. 2019).

Single nucleotide polymorphism (SNP) is a single base transversion or substitution, which occurs in more than 1% of the entire population. The SNP in the coding region may cause changes in the amino acid sequence, and these changes may remarkably alter the activity and biological characteristics of the encoded protein. Several reports have shown that polymorphisms of *HIF* gene can contribute to a variety of diseases. Huang et al. (Huang et al. 2018) found that *HIF-1α* C1772T TT genotype and *HIF-1α* G1790A AA genotype were involved in renal cell carcinoma susceptibility. Guo et al. (2015) showed that rs2057482 polymorphism in *HIF-1α* was associated with clinical outcome of Chinese aggressive hepatocellular carcinoma patients. In a Moscow population specifically, the IVS9-675C > A was prominently associated with the stroke risk (Tupitsyna et al. 2006). HIF-3α and HIF-1α have similar structures in the same family. We speculated that the polymorphisms of HIF-3α may be related to IS. However, no literature has reported the association between SNP of HIF-3α and IS risk. In order to verify our hypothesis, we conducted the case-control research to investigate whether these three SNP (rs3826795, rs3765609, rs2235095) in the HIF-3α gene were linked to IS risk in a Chinese population.

## Methods

### Study Population

The study subjects consisted of 310 IS patients and 302 controls. The patients with IS were continuously recruited from neurological department in Affiliated Hospital of Youjiang Medical University for Nationalities, Guangxi, China, from August 2016 to October 2019. The IS patients were ultimately diagnosed according to clinical simple symptom, examinations of nervous system, magnetic resonance imaging, or/and cranial computed tomography scan. These patients suffering from brain tumors, severe inflammatory diseases, and hereditary diseases were absolutely excluded from subjects of IS. The exclusion criteria for controls were as follows: history of stroke, tumorous, autoimmune diseases, genetic diseases, and cardiovascular diseases. A total of 302 gender- and age-matched healthy volunteers were eventually recruited after physical examinations in the same hospital during the same period. We collected the clinical data from medical record review of hospital, such as gender, age, smoking status, triglyceride (TG), total cholesterol (TC), apolipsprotein A1 (Apo-A1), apolipoprotein B (Apo-B) and high-density lipoprotein cholesterol (HDL-C), homocysteic acid (Hcy), and non-esterified fatty acid (NEFA). The study has been approved by our hospital ethics committee. Likewise, all subjects were unrelated Chinese, and they were selected continuously from the same geographical area.

### DNA Isolation and Genotyping

Genomic DNA was extracted from peripheral blood samples of each participant using a commercial kit (DP318, Qiangen, China). The primers of rs3826795, rs3764609, and rs2235095 were designed and synthesized by Shanghai Sangon Corporation. Three SNP sites were genotyped on the ABI 3500 Genetic Analyzer133 (CA, USA) using the custom-by-design 48-Plex SNPscan kit (G0104KS, Shanghai, China). Primer information is listed in Table 1. Moreover, a total of 10% of these samples were chosen at random to be examined by Sanger sequencing. Finally, we got a 100% consistent result.

**Table 1:** Primer information for genotyping assay of the *HIF-3α* gene

SNP	Length	Primer sequences(5′-3′)
Rs3764609		
F	20	TCTGCCTTGTACCCCAGACA
R	20	TCTGCCTTGTACCCCAGACG
Rs3826795		
F	24	GGAGACCCCTGAGCTGGATTGGTA
R	24	GGAGACCCCTGAGCTGGATTGATG
Rs2235095		
F	36	AGCTCAATCAATTAACGTTAACATCAATAAAACCTA
R	36	AGCTCAATCAATTAACGTTAACATCAATAAAACTTG

*HIF-3α* hypoxia-inducible factor-3α, *F* forward, *R* reverse

### Single Nucleotide Polymorphism Selection

We used the following criteria to select SNP: (1) tagSNPs in *HIF-3α* gene; (2) silico analysis predicted the potential functional SNP of promoter region in *HIF-3α* gene; (3) the frequency of secondary allele > 5% in Chinese Han population or candidate SNP sites previously reported in the literature. Eventually, three SNP were ascertained, including rs3826795, rs3764609, and rs2235095.

### Statistical Analysis

Continuous data were presented as mean ± standard deviation (SD) and compared using Student’s *t* test. Comparisons of categorical variables and Hardy-Weinberg equilibrium (HWE) were analyzed by *χ*^2^ test. Both odds ratios (ORs) and 95% confidence interval (CI) were calculated after adjustments for TG, TC, HDL-C, Apo-1, Apo-B, Hcy, and NEFA using logistic regression. Haplotype analysis and analysis of linkage disequilibrium (LD) were conducted on online SHEsis software (http://analysis.bio-x.cn/myAnalysis.php) (Yong and HE 2005). All statistical analysis is achieved through SPSS25 (version 25.0; SPSS, IL, USA). *P* < 0.05 was regarded really significant.

## Results

### Features of the Study Population

These clinical baseline characteristics are displayed in Table 2. The IS and control groups were not significantly different in sex, age, and smoking status (*P* = 0.68, 0.13, and 0.11, respectively). On the contrary, TG, TC, Apo-B, NEFA, and Hcy levels were significantly higher in IS patients, while HDL-C and Apo-A1 levels were lower in control groups (*P* all < 0.001).

**Table 2:** Clinical characteristics of the patients group and the control group

Variables	Controls, *n* = 302	Patients with IS, *n* = 310	*P* value
Age, year (mean ± SD)	62.2 ± 11.8	62.0 ± 11.3	0.68
Male/female	187 /115	210 /100	0.13
Smoking, yes/no	88/214	109/201	0.11
TC, mmol/L	4.62 ± 1.17	4.88 ± 0.94	0.001
TG, mmol/L	1.44 ± 1.37	1.82 ± 1.58	< 0.001
HDL-C, mmol/L	2.11 ± 10.45	1.13 ± 0.32	< 0.001
Apo-A1,g/L	1.76 ± 1.13	1.23 ± 0.26	< 0.001
Apo-B, g/L	0.75 ± 0.31	1.00 ± 0.31	< 0.001
Hcy, umol/L	13.6 ± 3.91	15.0 ± 3.84	< 0.001
NEFA, mmol/L	0.53 ± 0.28	0.71 ± 0.32	< 0.001

*IS*, ischemic stroke; *SD*, standard deviation; *TG*, triglycerides, *HDL*, high-density lipoprotein cholesterol; *TC*, total cholesterol; *TG*, triglyceride; *Apo-A1*, apolipsprotein A1; *Apo-B*, apolipoprotein B; *Hcy*, homocysteic acid; *NEFA*, non-esterified fatty acid

### Main Influence of *HIF-3α* Polymorphisms on IS Risk

Both genotypes and allele frequencies of the three SNP (rs3826795, rs2235095, and rs3764609) between two groups are displayed in Table 3. In the case and control group, genotype distribution did not deviate from HWE. The allele and genotype distribution of rs3826795 in two groups is presented in Fig. 2a and b. The AA genotype frequency of rs3826795 was 30.6% in the cases and 23.5% in the control groups (OR = 2.21, 95% CI, 1.10–4.44, *P* = 0.03). Moreover, rs3826795 AA genotype increased IS risk in AA vs GG+GA model analysis (OR 1.74, 95% CI = 1.02–2.97, *P* = 0.04). Moreover, the frequency of the rs3826795 A allele was 57.6% in IS and 42.4% in controls (OR = 1.48; 95% CI, 1.05–2.07, *P* = 0.02). By exploring the impact of 3 polymorphisms on IS risk, we found that the rs3826795 polymorphism significantly increased risk of IS. However, rs2235095 and rs3764609 sites were not different in the comparison between two groups.

**Table 3:** Association between *HIF-3α* polymorphisms and risk of IS

Polymorphisms	Controls, *n* = 302 (%)	IS, *n* = 310 (%)	Adjusted OR (95% CI)^a^	*P* value
Rs3826795				
GG	79 (21.2)	48 (15.3)	1.00	
AG	152 (50.3)	167 (53.4)	1.40 (0.75–2.61)	0.29
AA	71 (23.5)	95 (31.3)	2.21 (1.10–4.44)	0.03
AA+AG vs GG	223 (73.8)	262 (84.7)	1.64 (0.86–1.72)	0.10
AA vs AG + GG			1.74 (1.02–2.97)	0.04
G	310 (51.3)	263 (48.7)	1.00	
A	294 (42.4)	357 (57.6)	1.48 (1.05–2.07)	0.02
Rs3764609				
GG	45 (14.9)	44 (14.2)	1.00	
AG	160 (53.0)	150 (48.4)	0.91 (0.45–1.86)	0.80
AA	97 (32.1)	116 (37.4)	1.34 (0.64–2.79)	0.43
AA+AG vs GG			1.08 (0.55–2.12)	0.82
AA vs AG+GG			1.43 (0.87–2.35)	0.15
G	250 (41.4)	238 (38.4)	1.00	
A	354 (58.6)	382 (61.6)	1.22 (0.86–1.72)	0.26
Rs2235095				
GG	104 (34.4)	107 (34.5)	1.00	
AG	150 (49.7)	156 (50.3)	1.05 (0.62–1.78)	0.84
AA	48 (15.9)	47 (15.2)	1.06 (0.50–2.22)	0.89
AA+AG vs GG			1.06 (0.64–1.74)	0.84
AA vs AG+GG			1.02 (0.52–2.01)	0.95
G	358 (59.3)	370 (59.7)	1.00	
A	246 (40.7)	250 (40.3)	1.03 (0.73–1.46)	0.86

*CI*, confidence interval; *IS*, ischemic stroke; *OR*, odds ratio; *HIF-3α*, hypoxia-inducible factor-3α

^a^Adjusted by total cholesterol, triglyceride, high-density lipoprotein cholesterol, apolipoprotein A1, apolipoprotein B, homocysteic acid, and non-esterified fatty acid

### Haplotype Analysis

SHEsis was chosen to perform LD measurement and haplotype analysis, and the possible eight haplotypes are enumerated in Table 4. We observed that AGA was the main haplotype in IS and GGA was the main haplotype in controls (22.3% and 21.9%, respectively). Furthermore, we observed that the AGA haplotype may be associated with an increased risk of IS (OR 2.64, 95% CI 1.90–3.67, *P* < 0.001), while the GGA haplotype may be related to a reduced risk of IS (*P* < 0.05).

**Table 4:** Haplotype analysis in the patients with IS and the controls

	IS (%)	Controls (%)	OR (95% CI)	*P* value
AAA	81 (13.1)	49 (8.2)	1.68 (1.16–2.44)	0.01
AAG	57 (9.2)	63 (10.6)	0.86 (0.59–1.26)	0.44
AGA	138 (22.3)	59 (9.8)	2.64 (1.90–3.67)	< 0.001
AGG	80 (12.9)	120 (20.0)	0.59 (0.43–0.80)	< 0.001
GAA	61 (9.8)	111 (18.6)	0.48 (0.34–0.67)	< 0.001
GAG	50 (8.1)	20 (3.3)	2.59 (1.52–4.41)	< 0.001
GGA	101 (16.3)	131 (21.9)	0.70 (0.52–0.93)	0.01
GGG	51 (8.2)	46 (7.6)	1.08 (0.71–1.64)	0.72

*IS*, ischemic stroke; *OR*, odds ratio; 95% confidence interval

### Multiple Logistic Regression Analysis

IS risk factors were analyzed by logistic regression analysis. The results are shown in Table 5; the risk factors contained TG (OR = 1.02; 95% CI = 0.86–1.20, *P* = 0.85), TC (OR = 1.37; 95% CI = 1.08–1.73), HDL-C (OR = 1.00; 95% CI = 0.93–1.08), Apo-A1 (OR = 0.00; 95% CI = 0.00–0.01), Apo-B (OR = 52.76; 95% CI = 18.90–147.21), Hcy (OR = 1.10; 95% CI = 1.03–1.18), and NEFA (OR = 8.86; 95% CI = 3.52–22.32) (*P* all < 0.05). TC, Apo-A1, Apo-B, Hcy, and NEFA were still connected with IS risk after logistic regression analysis. However, after correction by comparisons, TG and HDL-C had no statistical significance. Therefore, further researches are necessary to confirm our results in larger sample sizes.

**Table 5:** Logistic regression analysis for identifying risk factors of IS

Variables	B	OR (95%CI)	*P* value
TG	0.02	1.02 (0.86–1.20)	0.85
TC	0.31	1.37 (1.08–1.73)	0.01
HDL-C	0.01	1.00 (0.93–1.08)	0.94
APO-A1	− 5.68	0.00 (0.00–0.01)	< 0.001
APO-B	3.97	52.76 (18.9–147.21)	< 0.001
Hcy	0.09	1.10 (1.03–1.18)	0.01
NEFA	2.18	8.86 (3.52–22.32)	< 0.001

*IS*, ischemic stroke; *TG*, triglycerides; *TC*, total cholesterol; *HDL*, high-density lipoprotein cholesterol; *Apo-A1*, apolipsprotein A1; *Apo-B*, apolipoprotein B; *Hcy*, homocysteic acid; *NEFA*, non-esterified fatty acid

### Association Between rs3826795 Polymorphism and Serum Hcy and NEFA Levels

**Fig. 1:**
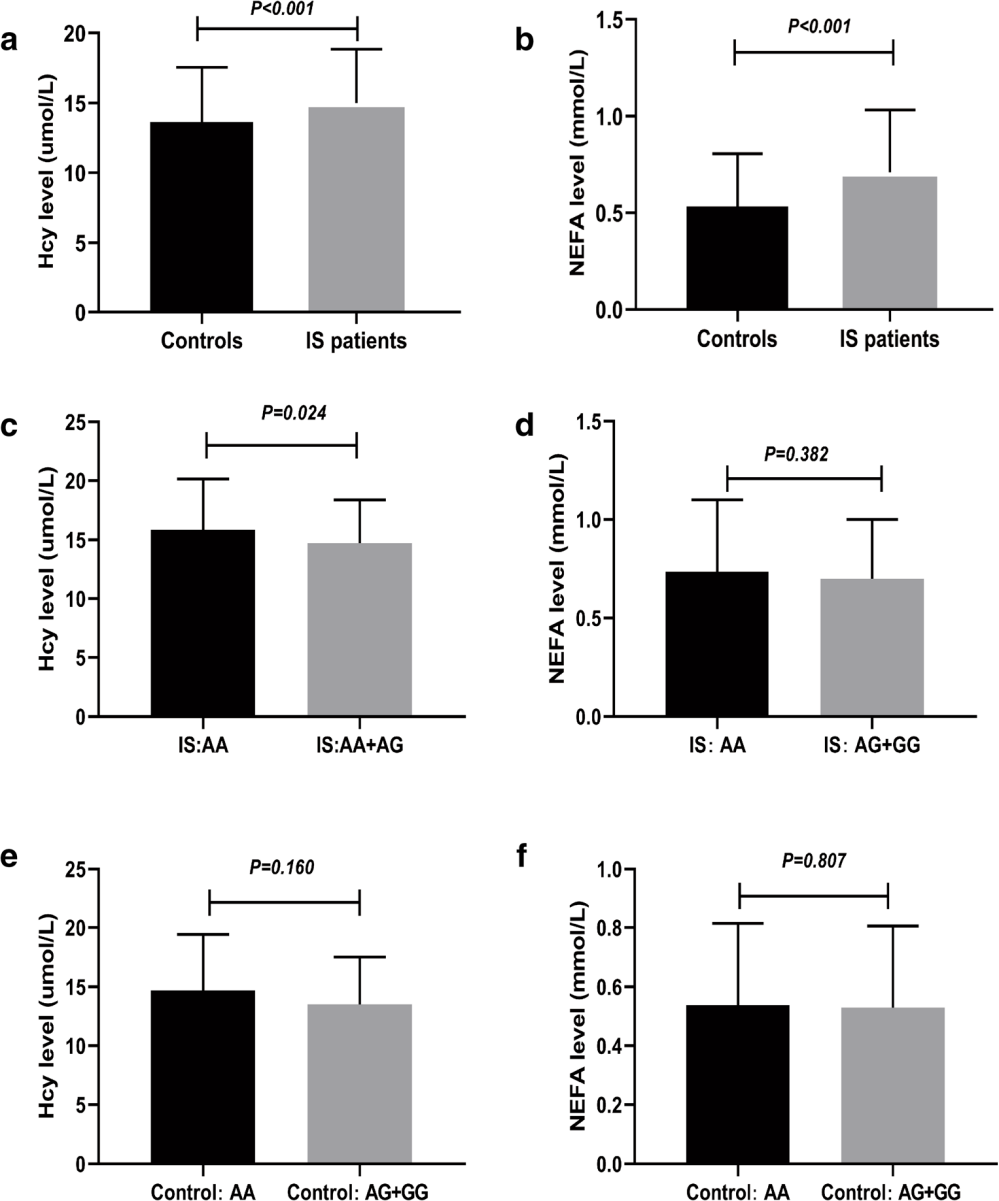
Association between rs3826795 polymorphism and levels of Hcy and NEFA. **a** An increased level of Hcy in IS patients compared to controls (*P* < 0.001). **b** Increased level of NEFA in IS patients compared to controls (*P* < 0.001). **c** Increased level of Hcy in IS patients carrying the rs3826795 AA compared to those carrying the rs3826795 AG+GG (*P* = 0.024). **d**–**f** The levels of Hcy and NEFA showed no significant differences among different genotype groups (*P* > 0.05). IS ischemic stroke, Hcy homocysteic acid, NEFA non-esterified fatty acid

We explored the association between serum levels of Hcy and NEFA and IS. As shown in Fig. 1a and b, compared to the control group, Hcy and NEFA levels in IS patients were significantly upregulated. Even using logistic regression analysis to adjust for common risks, such as the age, sex, smoking, TG, TC, HDL-C, Apo-A1, and Apo-B, Hcy and NEFA levels were still related to an increased risk of IS (Table 5) (*P* < 0.05). Additionally, we explored the association between polymorphism of *HIF-3α* rs3826795 and the levels of Hcy and NEFA. We found that patients with the rs3826795 AA genotype had higher levels of Hcy than those with AG+GG genotypes (*P* < 0.05). However, individuals with the AA genotype of rs3826795 in the control group had no significant difference in Hcy levels compared with the AG+GG control group (*P* = 0.160). Whether in the IS group or the control group, the NEFA levels of rs826795AA individuals were not statistically different from those of rs3826795 AG+GG individuals (*P* > 0.05). (Fig. 1c–f).

### Bioinformatics Analysis

**Fig. 2:**
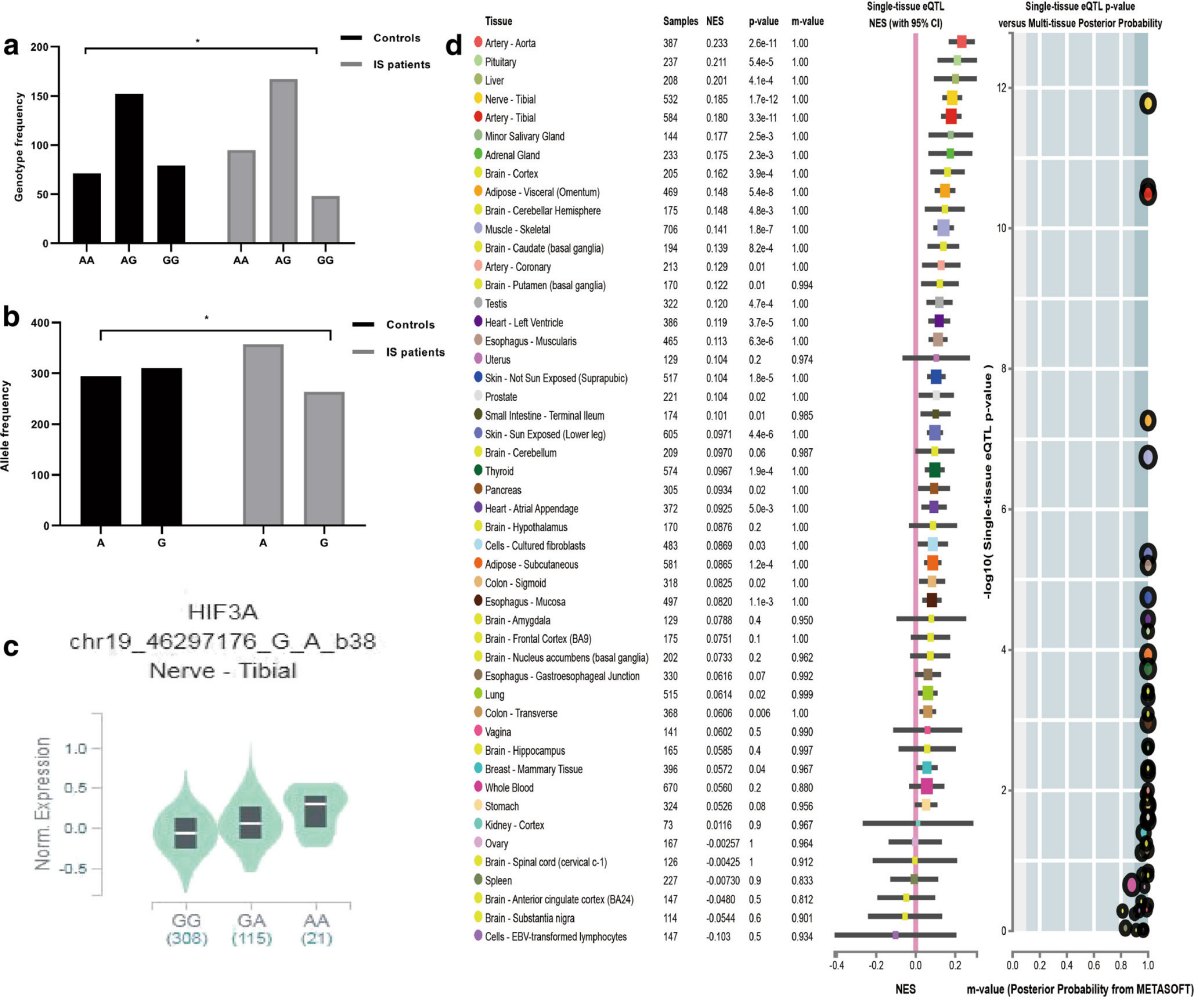
The rs3826795 AA genotype was associated with increased levels of *HIF-3α* compared to GG, *P* < 0.05 (C). Allele and genotype frequencies distribution of rs3826795 in control and IS patients (A, B), expression quantitative trait loci (eQTL) analysis of rs3826795 with gene expression in single tissue (D). *There were significant differences in genotype and allele frequencies of rs3826795 between two groups in Guangxi population (*P* < 0.01)

We obtained *HIF-3α* gene polymorphisms and tissue-specific expression in the GTEX database (https://www.gtexportal.org/home/). GTEX studied autopsy samples from healthy human donors. The healthy individuals carrying the rs3826795AA genotype increased the expression of *HIF-3α* (Fig. 2c). The analysis of the expressed quantitative trait locus (eQTL) suggested that the rs3826795 polymorphism is related to *HIF-3α* expression level in a single tissue (Fig. 2d).

## Discussion

In the current case-control study, we examined whether the SNP in *HIF-3α* affected IS risk. This is the first study to explore the relationship between rs3826795, rs3764609, and rs2235095 polymorphisms and IS risk. We found that the distributions of AA genotype and A allele of rs3826795 were significantly different between cases and controls. Further analysis found that AA genotype and A allele of rs3826795 were associated with an increased IS risk. Moreover, a dramatically increased level of Hcy and NEFA was found in IS patients compared with the control groups. Especially, IS patients carrying the rs3826795AA genotype had a higher level of Hcy than those with AG+GG genotypes. Our results indicate that rs3826795 in *HIF-3α* may be a risk biomarker related to the etiology of IS.

The *HIF* family has 3 distinct members from mammals to insects (*HIF-1/2/3*). HIF are involved in a wide range of biological processes, including vasomotor control, angiogenesis, energy metabolism, nucleotide metabolism, and cell proliferation and viability (Tekin et al. 2010). Numerous functional processes are linked to the pathogenesis of atherosclerosis. Genetic factors in the *HIF* gene may be associated with atherosclerosis (Jain et al. 2018) and inflammation, which are linked to the occurrence of IS (Cheng et al. 2014; Cole et al. 2008; Davis et al. 1987). Karshovska and his colleagues found that *HIF-1α* could increase atherosclerosis by necrotic core formation (Karshovska et al. 2020). Furthermore, other researchers found that in the process of atherosclerosis, *HIF-1* has an effect on macrophages and vascular cells, which is used as a target for treating atherosclerosis (Jain et al. 2018). However, as a member of *HIF* family, the *HIF-2* had a protective effect on atherosclerosis by inhibiting adipose, plasma ceramide, and plasma cholesterol levels (Zhang et al. 2019). Taken together, *HIF- (1, 2, 3)* belongs to same family. *HIF-1α* and *HIF-2α* are strongly linked to transcriptional regulation of *HIF-3α*. The evidence demonstrated that the *HIF-3α* may play a key role in IS, which may be a therapeutic target for this disease.

In the study, our experiment confirmed that *HIF-3α* polymorphism and IS risk were related. As for rs3826795, to our knowledge, the relationship between rs3826795 and IS was revealed for the first time. We observed that the rs3826795 AA genotype had a 2.21 times elevated IS risk. Meanwhile, our results indicated the A allele is closely related to an increased risk of IS. Additionally, the A-G-A haplotype may cause the susceptibility of IS. These differences were significant even after adjusting for potential confounding factors (i.e., TC, TG, HDL-C, Hcy, and NEFA). In addition to common factors, risk factors of IS included atherosclerosis as well as obesity (Aa and Samiee 2009; Bhupathiraju and Hu 2016; Carl et al. 2014). A research by Wang et al. (2017) showed that the *HIF-3α* rs3826795 polymorphism interacted with ALT of obesity, and ALT elevation was closely related to central adiposity and related features including hypertension and dyslipidemia. Huang et al. (2015) reported that DNA methylation of *HIF-3α* rs3826795 interacted with total B vitamins in relation to BMI changes. Rausch and his team (Johnson et al. 2012) found that obesity was relevant to increased HIF expression in male C57BL/6 J mice. The evident indicates that the *HIF-3α* may play a vital role in IS. Interestingly, rs3826795 is located in the first intron of *HIF-3α*, and is a noncoding region of *HIF-3α*. However, there is still an association between rs3826795 and IS. The possible reason is that introns can dramatically influence expression of gene, such as containing enhancer or increasing mRNA accumulation (Moabbi et al. 2012; Rose 2008). The structure of human *HIF-3α* has a forceful commonality with Zebrafish *HIF-3α* (Zhang et al. 2012). Hypoxia and ischemia significantly increase *HIF-3α* levels in the liver, heart, brain, and ovary. Yet, hypoxia did not elevate *HIF-3α* levels in the kidney, gill, and testis. Heidbreder et al. (2003) suggested that hypoxia increased *HIF-3α* but not *HIF-1α* and *HIF-2α* levels in the hippocampus and cerebral cortex, rat’s lung. Hence, *HIF-3α* mRNA expression regulated by hypoxia and ischemia is tissue-specific. Yan et al. (Jun Yan et al. 2014) reported that *HIF-3α* expression in whole blood cell was upregulated after acute IS. In addition, the GTEx database showed that the rs3826795 had differences in *HIF-3α* expression (Fig. 2d). We observed that the AA genotype frequency in IS was clearly higher than that in controls. The GTEx database also showed that subjects carrying the rs3826795 AA genotype had higher levels of HIF-3α expression (Fig. 2c). Further mechanism experiments are needed to verify the connection between rs3826795 SNP and HIF-3α expression. In a word, the important role of rs3826795 in HIF-3α may be considered as a novel target for treating IS.

In this present study, we also studied the relationship between Hcy or NEFA and IS risk. Compared with the controls, the Hcy and NEFA serum levels among IS patients were significantly upregulated. Serum NEFA and Hcy were associated with atherosclerosis and IS formation, and high levels of Hcy and NEFA were correlated to poor prognosis in IS patients (Jickling and Spence 2014; Wei et al. 2019). Wei (Wei et al. 2019) found that serum Hcy levels were upregulated in IS patients, especially those carrying rs2666433 AA genotype. Similar to Wei’s findings, further comparing the difference in Hcy and NEFA expression between rs3826795 AA and AG+ GG, we found that IS patients with rs3826795 AA had higher levels of Hcy. We speculated that rs3826795 AA may increase the risk of IS by upregulating the levels of Hcy. Further experiments are needed to explore the potential molecular mechanism of *HIF-3α* rs3826795 affecting the occurrence of IS.

The current research has several limitations. Hospital-based cases and controls cannot eliminate selection bias. The involvement of environmental factors in the development of IS has been widely accepted. Due to the lack of objective data, we cannot assess the impact of gene-environment interaction. The same polymorphism varies among different races. Therefore, the results we got do not directly apply to all races. Perhaps, once these limitations are eliminated, we can have a better and more comprehensive understanding of these SNPs in the development of IS.

## Conclusion

In conclusion, we reported that rs3826795AA polymorphism in the *HIF-3α* may be associated with the susceptibility of IS for the first time. These findings suggest that the rs3826795 may be an underlying biomarker for the occurrence and development of IS. A larger sample size is required in various ethnic groups. Further researches are crucially important to understand biological function of the rs3826795 in the development of IS.

## Data Availability

The raw datasets generated and/or analyzed during the current study are not publicly available in order to protect participant confidentiality.
